# Visual grading characteristics and ordinal regression analysis during optimisation of CT head examinations

**DOI:** 10.1007/s13244-014-0374-9

**Published:** 2014-12-16

**Authors:** Francis Zarb, Mark F. McEntee, Louise Rainford

**Affiliations:** 1 Department of Radiography, Faculty of Health Sciences, University of Malta, Msida, Malta; 2 Discipline of Medical Radiation Sciences and Brain and Mind Research Institute, Faculty of Health Sciences, The University of Sydney, Sydney, Australia; 3 School of Medicine & Medical Science, Health Science Centre, University College Dublin, Belfield, Dublin 4, Ireland

**Keywords:** CT, Optimisation, Image quality, Visual grading characteristics, Ordinal regression

## Abstract

**Objectives:**

To evaluate visual grading characteristics (VGC) and ordinal regression analysis during head CT optimisation as a potential alternative to visual grading assessment (VGA), traditionally employed to score anatomical visualisation.

**Methods:**

Patient images (n = 66) were obtained using current and optimised imaging protocols from two CT suites: a 16-slice scanner at the national Maltese centre for trauma and a 64-slice scanner in a private centre. Local resident radiologists (n = 6) performed VGA followed by VGC and ordinal regression analysis.

**Results:**

VGC alone indicated that optimised protocols had similar image quality as current protocols. Ordinal logistic regression analysis provided an in-depth evaluation, criterion by criterion allowing the selective implementation of the protocols. The local radiology review panel supported the implementation of optimised protocols for brain CT examinations (including trauma) in one centre, achieving radiation dose reductions ranging from 24 % to 36 %. In the second centre a 29 % reduction in radiation dose was achieved for follow-up cases.

**Conclusions:**

The combined use of VGC and ordinal logistic regression analysis led to clinical decisions being taken on the implementation of the optimised protocols. This improved method of image quality analysis provided the evidence to support imaging protocol optimisation, resulting in significant radiation dose savings.

***Main Messages*:**

• *There is need for scientifically based image quality evaluation during CT optimisation*.

• *VGC and ordinal regression analysis in combination led to better informed clinical decisions*.

• *VGC and ordinal regression analysis led to dose reductions without compromising diagnostic efficacy*.

## Introduction

Producing high-quality images in computerised tomography (CT) is important for image interpretation to ensure that the maximum diagnostic information is available to facilitate the visualisation of discrete changes in anatomy indicating early pathological processes [1–3]. Higher quality CT images, however, normally imply a higher radiation dose to the patient since changes in scan parameters are required to facilitate high resolution [4]. The standard of image quality mainly depends on the preferences of radiologists and their willingness to balance low-noise, high-quality CT images with the impact upon radiation levels administered. Noise is a major factor in determining acceptable image quality and often dictates the radiation dose for a particular CT protocol. Increases in noise degrade both low-contrast resolution and spatial resolution and therefore influence the radiologists’ perception of the image [5–8]. However, an increase in noise up to certain levels may not necessarily impair the image diagnosis [9, 10].

The aim of image quality optimisation is to provide an image that is suitable for the clinical task with the lowest radiation dose given to the patient. ‘*It is whether the clinical information required is contained in the image and can be interpreted by the observer that is important rather than whether the appearance of the image is pleasing to the eye*’ [11]. The threshold of image quality should be one able to deliver enough information to the radiologist to permit a medical decision to be taken with an acceptable amount of assurance [11].

Hence, there is a need to have a keen understanding of image quality evaluation tools and methods of data analysis to identify the required level of image quality required for diagnosis in the development of optimised scan protocols using the lowest possible radiation dose. Effective and scientifically accepted methods of assessing image quality are needed for the implementation of such optimised imaging protocols across all imaging modalities including CT [1–3, 12–15].

To limit any uncertainties in interpretation, observer performance tests on images obtained using CT scanning protocols should be carried out testing visualisation of anatomical structures or known pathologies. The usefulness of observer performance studies where observers visually grade image quality is attributed to the following characteristics [16]:

- The validity of such studies is assumed to be high since the observer’s ratings take into account all technical factors in reproducing anatomical structures on the image together with the experience and confidence of the observer in identifying and interpreting the image.
- Image assessment is based on the visualisation of clinically relevant anatomical structures using established standards such as the European guidelines on quality criteria.
- The studies are easy to conduct following a clear and reproducible methodology and take practical consideration of radiology availability increasing the chances for participation.

Observer performance methods such as image criteria (IC) studies, visual grading analysis (VGA) and receiver-operating characteristic (ROC) analysis are now established methods for the analysis of image quality. IC and VGA are useful in the majority of cases where the patient examinations present as normal anatomy [17]. ROC is of value for the identification and location of pathologies [18].

CT examinations producing anatomical structures obtained from either anthropomorphic phantoms or animal models make it easier to produce large numbers of images with no concern about the ethical issues associated with the irradiation of patients. Results of such studies facilitate the comparison of optimised protocols with current ones prior to their implementation on patients in the clinical setting [13, 19–21].

Established methods in performing observer and diagnostic performance tests make it possible to measure image quality by the evaluation of anatomical structures seen on the CT images against a set of criteria that have to be fulfilled [21–24]. Visual grading analysis (VGA) facilitates the quantification of subjective opinions and involves grading of the visibility of anatomical structures on the images. In relative VGA, the visibility of anatomical structures is compared and graded against the visibility of the same structures within a reference image. The observers grade the visibility of the structure with an arbitrary ordinal scale where ‘0’ implies a visibility equal to the structure within the reference image, while negative or positive values imply inferior or superior visibility respectively. In absolute VGA, the visibilities of anatomical structures within the images are graded against each other. The scales are ordinary and are usually given a description facilitating interpretation and improving the agreement between observers. A VGA score calculated from the results of such analysis allows statistical analysis of the differences [13, 21].

In 2007 Bath and Mansson indicated the inappropriate analysis of visual grading data using parametric tests and recommended a novel method of analysing such data called Visual Grading of Characteristics: VGC analysis. VGC treats the scale steps as ordinal with no assumptions on the distribution of the data [25]. The resemblance between VGC and receiver-operator characteristic (ROC) analysis leads to the possibility of using the well-established ROC evaluation methods in analysing VGC data. The variation in the visual grading of the reviewers of two imaging techniques can be used to describe the variation between the two techniques in the same way as in an ROC study. VGC can be considered as a repeated image criteria scoring, where reviewers change their threshold for the necessity of fulfilling each criterion in a similar way to the scale steps in an ROC study. The reviewers therefore state their confidence concerning the fulfilment of a criterion obtaining an ordinal scale. As in ROC analysis, the different ratings do not necessarily correspond to the same numerical intervals on the decision scale nor do all reviewers use the ratings with the same meaning since the ordinal scale is just used to test the probability distribution for each imaging technique [25].

In 2010, Smedby and Fredrikson proposed a method for analysing ranked visual grading data using ordinal logistic regression analysis [26]. Ordinal logistic regression is a statistical technique able to process data on an ordinal scale that handles situations involving several factors that could potentially influence the outcome [27, 28]. Scott (1997) states that a lack of a full review of ordinal data increases the potential for lost information and that ordinal regression facilitates the analysis whilst taking account of a number of explanatory variables, accounting for the effects of each in the form of an odds ratio, so excluding unverifiable assumptions [27]. In 2011, Smedby presented a study quantifying potential radiation dose reduction with visual grading regression at the Medical Imaging Perception Society’s (MIPS) 14th Conference held in August 2011 in Dublin, Ireland [26]. This study was published in the British Journal of Radiology (BJR) in 2012 [29]. Although ordinal logistic regression analysis is an established statistical method for analysing ordinal data, only recently have publications specifically recommended its use in the investigation of diagnostic efficacy [26].

Effective and scientifically accepted methods of assessing image quality are needed for the clinical implementation of optimised CT protocols [1–3, 12–15]. This study investigated image quality evaluation using VGC during optimisation of CT examinations of the head. Image quality scores obtained from this visual grading assessment were analysed using VGC and ordinal regression analysis and the impact of their findings upon the achieved radiation dose savings during the review of the optimised scanning protocols. Detail of the optimisation process for the Maltese data set was previously published [30, 31].

## Materials and methods

Ethical approval was obtained from the clinical centres and from the governing ethics institution (UREC Ref No: 001/2009).

### Image data set: collation and review

This research group had previously identified CT examinations of the head (43 %) as the most commonly requested and performed CT examination in Malta [21]. Head CT examinations using the locally optimised protocols were performed in each participating CT suite (n = 2): a GE BrightSpeed Elite16-slice CT scanner at a public hospital with an annual CT referral rate of 6,460 head CT examinations and a Philips Brilliance 64-slice CT scanner in a private centre with an annual CT referral rate of 300 head CT examinations. The resulting 66 CT data sets for image quality evaluation included the current protocol (n = 30), optimised protocol (n = 30) and duplicate examinations (n = 6) to facilitate inter- and intra- reviewer reliability.

The coded CT data sets were reviewed by six local resident radiologists including: two consultant radiologists, one with more than 20 years’ experience in CT reporting and the second having more than 5 years, two senior radiology registrars, both with more than two years’ experience and two radiology trainees. These images were presented using ViewDex, a Java-based software for presentation and evaluation of medical images in reviewer performance studies developed at Sahlgrenska University Hospital, Goteborg University and Sodra Alvsborg Hospital [32].

The images were displayed on primary monitors using a General Electric (GE) Advantage Workstation (AW) v.4.3_07, previously tested and satisfying the recommendations by the American Association of Physicists in Medicine (AAPM) as outlined in task group 18 [33], having 3-megapixel monitors (1,536 * 2,048 pixels) driven by a BarcoMed Coronis graphics card, with a maximum luminance of 725.22 cd/m^2^ [34]. Ambient lighting levels adhered to AAPM recommendations for diagnostic reading workstations (15–60 lux) [33, 35] and were measured with a calibrated Unfors Light-O-Meter photometer (Billdal, Sweden).

The radiologists declared their confidence for each of the EU CT anatomical criteria [36] using a five-point scale [16] as follows:

- 1: confident that the criterion is not fulfilled;
- 2: somewhat confident that the criterion is not fulfilled;
- 3: indecisive whether the criterion is fulfilled of not;
- 4: somewhat confident that the criterion is fulfilled;
- 5: confident that the criterion is fulfilled.

The image data sets predominantly consisted of normal cases. Clinical indications included head trauma, cerebrovascular accident (CVA) or stroke and headaches. Eight cases presented abnormal findings: four subdural haematomas and four infarcts, all classified as obvious pathologies by a consultant radiologist. The pathologies were deemed not to distort the anatomy indicated in the evaluation criteria. VGC curves and ordinal regression analysis were employed to analyse the data.

### Quantification of dose reduction recorded between systems

The overall reductions in radiation dose measured in terms of radiation dose quantities: volume CT dose index (CTDIvol) and dose length product (DLP) following optimisation are summarised in Table 1. CTDIvol is the main radiation dose indicator in spiral CT, integrating the radiation dose for a single slice delivered both within and beyond the scanned volume, representing the average radiation dose for a single slice in the scanned volume for contiguous scans. CTDIvol given in milligrays (mGy) is an accurate indicator of the radiation dose per slice within the scanned volume [1, 36–39]. The CTDIvol multiplied by the total scan length in centimetres is given as the dose length product (DLP) given in milligray cm (mGy cm) [2, 36–38, 40]. DLP correlates better with the patient radiation dose than CTDIvol and can easily be used as an indicator of the given radiation dose. There is a linear relationship between DLP and radiation dose and a linear relationship between radiation dose and the stochastic risk. Hence, DLP can be used to compare the stochastic risk between different CT examinations [41]. DLP is a more realistic indicator of the radiation dose and, in calculating the DLP, the measure of CTDIvol is still required. While CTDIvol is estimated based on the selected scan parameters prior to imaging, DLP is calculated after the examination has taken place.

**Table 1:** Comparison of mean CT doses between current and optimised protocols (confidence interval p < 0.05)

CT head	GE BrightSpeed			Philips Brilliance		
Independent samples t-test	Current	Optimised	p	Current	Optimised	p
	n =24	n =20		n =24	n =20	
CTDIvol (mGy)	35.8	33.1	0.00	39.6	28.3	0.00
(Range)	(30.0-37.6)	(29.8-36.1)		(39.6-39.6)	(28.3-28.3)	
DLP (mGy-cm)	489.4	461.5	0.03	694.8	637.3	0.03
(Range)	(420.0-564.0)	(389.2-575.1)		(491.0-820.0)	(541.1-823.1)	

#### Data analysis

Image quality scores obtained from the evaluation of the image data sets by local radiologists were analysed using VGC. VGC analysis is performed in three steps:

1. A frequency table (2 × n frequency table, where n = number of categories) summarises the results for the two scan protocols separately.
2. The VGC data points in the frequency table represent the coordinates of the VGC curve. As in an ROC curve the origin of a VGC curve per definition is “0”. The data points are arranged according to the cumulative (relative) frequencies of the corresponding categories. The last point includes all decisions and therefore is “1” [16].
3. The VGC points are plotted to produce a VGC curve indicating the sensitivity or true positive fraction (TPF). The curves are produced using the same software for obtaining ROC curves [13]. The VGC curves for this study were created using the ROC analysis web-based calculator for ROC curves developed by John Eng, M.D., and Russell H. Morgan at the Department of Radiology and Radiological Science, Johns Hopkins University, Baltimore, MD, USA (available online at: http://www.rad.jhmi.edu/jeng/javarad/roc/JROCFITi.html).

The area under the curve (AUC_VGC_) can be considered as a measure or accuracy index of the difference in image quality between the two techniques. A VGC curve situated on or near the diagonal (AUC_VGC_ =0.5) indicates that the two scan protocols produce identical image quality. The greater the AUC_VGC_ (>0.5) indicates better image quality for the scan protocol on the vertical axis of the plot [16, 25].

Image quality scores together with radiation dose measurements and protocol type were also analysed using ordinal regression analysis. Ordinal logistic regression analysis takes into account potential confounders of the association between two cohorts: the independent variables and the end result (dependent variable), with the measure of association being the odds ratio [42]. In this study, random effects include the patients and the radiologists participating in the study while the independent variables are the scan protocols and the radiation dose administered as the DLP. The dependent variable is the rating scores as an indication of image quality. The ordinal logistic regression model (proportional odds model) is the appropriate model for analysing rating scores since these are ordinal categorical responses.

Ordinal regression analysis was performed using the IBM Statistical Package for Social Sciences (SPSS version 19), where differences were considered significant outside the 95 % confidence interval (p ≤ 0.05).

## Results

### Image quality evaluation: VGC

**Fig. 1:**
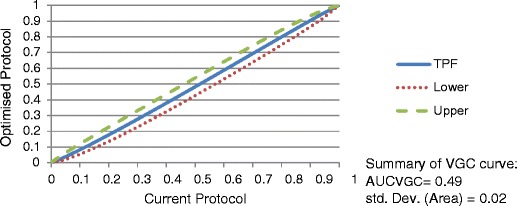
VGC curve—GE BrightSpeed

**Fig. 2:**
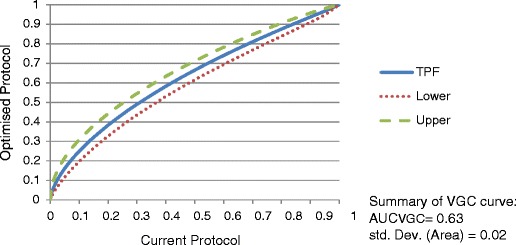
VGC curve—Philips Brilliance

VGC curves (Figs. 1 and 2) indicate that the optimised protocols do not differ significantly from the current protocols in terms of image quality. A one-sample t-test on the values of the area under the curve (AUC_VGC_) (Table 2) demonstrated no significant difference from the 0.5 value (p ≥ 0.05) despite radiation dose reductions.

**Table 2:** AUC_VGC_ results of one-paired sample t-test

Head	Test value =0.5									
	N	Mean	SD	SE mean	t	df	p-value	Mean difference	95 % confidence interval of the difference	
Malta AUC_VGC_	4	0.56	0.06	0.03	1.89	3	0.16	0.06	−0.04	0.15

### Image quality evaluation: ordinal regression analysis

Ordinal regression analysis was applied to further identify differences between the protocols and their effect on the quality of the images produced and therefore as predictors of image quality.

Overall results presented in Table 3 show that DLP and criteria have no significant impact on the rating scores (p ≤ 0.05). The protocol used however does have a significant impact on the quality of the images in terms of rating scores (p ≤0.05). The difference in the protocols is based on the imaging parameters selected with the optimised protocol administering a significantly lower radiation dose (Table 1).

**Table 3:** Overall results: ordinal regression analysis

Omnibus test			
	Likelihood ratio Chi-square	df	p-value
Philips Brilliance	170.19	6	0.00
GE BrightSpeed	310.00	6	0.00
Tests of model effects			
Type III			
Source	Wald chi-square	df	p-value
Philips Brilliance			
DLP	1.25	1	0.26
Criteria	1.25	1	0.26
Protocol	44.53	1	0.00
GE BrightSpeed			
DLP	0.02	1	0.90
Criteria	11.81	1	0.00
Protocol	278.47	4	0.00

Dependent variable: rating score

Model: (Threshold), DLP, criteria, protocol

Results presented in Table 4 indicate that only criteria 2 (*visually sharp reproduction of the basal ganglia*) had a negative odd for the GE BrightSpeed, while criteria 1 (*visually sharp reproduction of the border between white and grey matter*) and again criteria 2 had a negative odd for the Philips Brilliance. For every one-unit increase, the odds that the selected protocol is the current protocol rather than the optimised protocol increase by: *(Odds Ratio (B) – 1) × 100* [42].

**Table 4:** Criteria results: ordinal regression analysis

	Parameter estimates						
			95 % Wald confidence interval		Hypothesis test		
Parameter	Odds (B)	S E	Lower	Upper	Wald chi-square	Df	p-value
GE BrightSpeed							
Threshold [rating score = 1]	−1.87	0.57	−2.98	−0.75	10.73	1	0.00
[Rating score = 2]	−0.60	0.57	−1.71	0.51	1.12	1	0.29
[Rating score = 3]	0.44	0.57	−0.67	1.55	0.61	1	0.44
[Rating score = 4]	1.46	0.57	0.35	2.58	6.62	1	0.01
DLP	0.00	0.00	−0.00	0.00	0.02	1	0.90
[Criteria = 1]	0.61	0.20	0.23	1.02	9.42	1	0.00
[Criteria = 2]	−1.17	0.15	−1.46	−0.87	60.80	1	0.00
[Criteria = 3]	0.95	0.15	0.65	1.24	39.22	1	0.00
[Criteria = 4]	0.38	0.15	0.09	0.67	6.622	1	0.01
[Criteria = 5]	0.00	.	.	.	.	.	.
[Protocol = 1 ]	0.35	0.1005	0.15	0.54	11.81	1	0.00
[Protocol = 2 ]	0	.	.	.	.	.	.
(Scale)	1						
Philips Brilliance							
Threshold [rating Score = 1]	−4.46	0.56	−5.56	−3.36	63.45	1	0.00
[Rating score = 2]	−2.47	0.53	−3.50	−1.44	22.01	1	0.00
[Rating score = 3]	−1.16	0.52	−2.18	−0.14	4.97	1	0.03
[Rating score = 4]	1.28	0.52	0.26	2.29	6.01	1	0.01
DLP	−0.00	0.00	−0.00	0.00	1.25	1	0.26
[Criteria = 1]	−1.02	0.20	−1.42	−0.62	24.86	1	0.00
[Criteria = 2]	−1.37	0.20	−1.77	−0.10	45.34	1	0.00
[Criteria = 3]	0.62	0.20	0.23	1.02	9.42	1	0.00
[Criteria = 4]	0.03	0.20	−0.37	0.43	0.02	1	0.90
[Criteria = 5]	0.00	.	.	.	.	.	.
[Protocol = 1 ]	0.86	0.129	0.61	1.12	44.53	1	0.00
[Protocol = 2 ]	0	.	.	.	.	.	.
(Scale)	1						

Dependent variable: rating score model: (Threshold), DLP, criteria, protocol

The kappa (k) values for resident radiologists (n = 6) ranged between 0.33 and 0.59 showing fair to moderate agreement in their interpretation of the repeated scans, while the p-value was ≤0.05, implying significant agreement between the two interpretations of the repeated scans. Cronbach’s alpha (α) measured 0.76, indicating an acceptable level of internal consistency within the local resident radiologist, with inter-reviewer correlations ranging between 0.26 and 0.45.

## Discussion

The advantages of using visual grading studies in the evaluation of clinical images are that these can be carried out with clinically available images and there is no need for a gold standard during the evaluation. However the use of appropriate data analysis methods should be emphasised. The terminology in this field is somewhat confusing as some authors use VGA to denote standard statistical tests with assumptions that may not be appropriate since the data in a VGA str ordinal. Analysing visual grading analysis methods with parametrical statistical tests such as t-tests and analysis of variance (ANOVA) incorrectly assumes the grading data are an interval variable; VGC and ordinal regression analysis correctly treat visual grading data as ordinal and categorical [29].

An added advantage of the logistic regression model is that it can simultaneously consider multiple factors influencing the quality of the image. The inclusion of multiple factors in the logistic statistical model means that more complete detailed information can be obtained [27, 28]. Based on this information a specific clinical decision can be taken.

The findings of the VGC and ordinal regression analysis also led to consultation with participating resident radiologists highlighting the importance of image quality criteria for CT head imaging, which could be weighted differently depending on the pathology being investigated. Asked to rate the five criteria in order of importance, they concluded that criterion 1 (*visually sharp reproduction of the border between white and grey matter*) and criterion 5 (*visually sharp reproduction of the cerebrospinal fluid space over the brain*) are the two most important. The majority of brain pathologies such as infarcts and brain tumours require image quality levels that allow differentiation between these structures. In the case of suspected fresh haemorrhage, since it is hyperdense in relation to the surrounding brain tissue, a lower image quality may be sufficient for diagnosis. No specific criteria were indicated for white matter disease as this is normally determined by MRI.

Based on the results of both VGC and ordinal regression analysis performed in this study, the optimised protocols were implemented for all patient presentations (inclusive of trauma) for general brain on the GE BrightSpeed scanner as the negative odds did not affect any of the two most important criteria. However, the optimised protocol was limited to follow-up cases on the Philips Brilliance as criteria 1 findings were affected by the optimisation process and this was considered important for initial diagnosis by the radiology experts.

While low-dose CT has been shown to be viable in high contrast imaging [6], it is still unclear whether the same radiation dose reductions are possible in areas of low contrast differences such as intracranial brain structures. Initial CT examinations are mainly targeted for the diagnosis of subtle changes in intracranial structures such as lacunar infarcts requiring optimal contrast resolution and therefore it may not be appropriate to use low-dose, high-noise scan protocols [43]. Follow-up CT examinations on the other hand are performed with the purpose of identifying gross morphological changes involving structures with high contrast or large structures and can therefore benefit from the use of radiation dose reduced protocols especially if performed for repeat patient imaging. The indications of follow-up brain scans are frequently gross imaging findings that may change or affect the clinical management of the patient. Examples include follow-up for traumatic or non-traumatic haemorrhage, raptured aneurysms, stroke or evaluation of ventricular size in cases of hydrocephalus [6, 43, 44].

A limitation of this study is that image quality evaluation was based primarily on morphologically normal anatomical structures. Radiologists were not asked to evaluate or comment on any pathology present in the image data sets. So the question still remains as to whether this low radiation dose is applicable for specific brain pathologies, which frequently present as low contrast differences in comparison to normal brain tissue [6, 43–45]. The inclusion of pathologies together with an ROC analysis to investigate the applicability of the optimised protocols in the diagnosis of subtle to obvious pathologies is recommended. Consideration of different weighting levels for anatomical criteria is suggested by the authors to be incorporated in future image quality research.

The absence of a significant finding may be related to an insufficient number of observations rather than as a result of the statistical approaches applied; however the number of observations involving six expert readers and 66 CT data sets pre and post optimisation aligns with similar observer studies and exceeds several [46–49]. Additionally, as the CT images were of the brain, the variability in subject matter was minimal with respect to anatomical criteria due to patient size differences compared to studies that have looked at other anatomical regions such as the chest or abdomen. It is therefore suggested that the findings are representative of the statistical tool employed. Currently research incorporating both VGC and ordinal regression analysis in review of clinical images and anatomical criteria is limited and therefore further research involving these statistical test tools is recommended.

The use of image quality criteria facilitates the use of visual grading studies. European guidelines on quality criteria for computed tomography (EUR 16262) list anatomical structures for the visualisation for specific CT examinations such as the cranium (general brain and skull base); face and neck (face, sinuses, petrous bone, orbits, sella, salivary glands, pharynx and larynx); spine (vertebral and paravertebral structures, lumbar spine, disc herniation and spinal cord); chest (general chest, mediastinal vessels and high resolution CT); abdomen and pelvis (general abdomen, liver, spleen, kidneys, pancreas, adrenal glands and general pelvis); bones and joints (pelvis and shoulder) [36]. Additional research is recommended to encompass the range of CT examinations indicated in the European guidelines with increased focus upon anatomical structural criterion definition and the weighting of criteria with respect to the diversity of patient presentation.

## Conclusion

This work has confirmed the utility of VGC and ordinal regression during optimisation of radiation dose and image quality in CT. These are valid statistical methods for the data generated during VGA experiments. The use of this method should be encouraged over statistical tests for VGA that assume normality or continuous data.
